# Reflectance structured illumination imaging of internalized cerium oxide nanoparticles modulating dose-dependent reactive oxygen species in breast cancer cells

**DOI:** 10.1016/j.bbrep.2020.100745

**Published:** 2020-02-14

**Authors:** Melissa Do, Kayla Stinson, Remo George

**Affiliations:** Department of Clinical & Diagnostic Sciences, University of Alabama at Birmingham, Birmingham, AL, 35294, USA

**Keywords:** Cerium oxide nanoparticles, Reflectance structured illumination imaging, Radiosensitizer, Nanoparticle imaging, Nanoparticle uptake, Reactive oxygen species

## Abstract

Cerium oxide nanoparticles have been shown to sensitize cancer cells to radiation damage. Their unique redox properties confer excellent therapeutic potential by augmenting radiation dose with reactive oxygen species mediating bystander effects. Owing to its metallic properties, cerium oxide nanoparticles can be visualized inside cells using reflected light and optical sectioning. This can be advantageous in settings requiring none or minimal sample preparation and modification. We investigated the use of reflectance imaging for the detection of unmodified nanoceria in MDA MB231 breast cancer cells along with differential interference contrast imaging and fluorescent nuclear labeling. We also performed studies to evaluate the uptake capability, cellular toxicity and redox properties of nanocaria in these cells. Our results demonstrate that reflectance structured illumination imaging can effectively localize cerium oxide nanoparticles in breast cancer cells, and when combining with differential interference contrast and fluorescent cell label imaging, effective compartmental localization of the nanoparticles can be achieved. The total number of cells taking up the nanoparticles and the amount of nanoparticle uptake increased significantly in proportion to the dose, with no adverse effects on cell survival. Moreover, significant reduction in reactive oxygen species was also observed in proportion to increasing nanoceria concentrations attesting to its ability to modulate oxidative stress. In conclusion, this work serves as a pre-clinical scientific evaluation of the effective use of reflectance structured illumination imaging of cerium oxide nanoparticles in breast cancer cells and the safe use of these nanoparticles in MDA MB231 cells for further therapeutic applications.

## Highlights

• Internalized cerium oxide nanoparticles are imaged with reflected light in breast cancer cells for the first time.
• Cerium oxide nanoparticles demonstrated no toxicity in MDA MB231 breast cancer cells
• Cerium oxide nanoparticles modulated free radicals in MBA MB231 cells in a dose dependent manner

## 1 Introduction

Nuclear medicine and radiation therapy are among the most effective cancer treatments available and more than half of the cancer patients undergo ionizing radiation therapy owing to its ability to eradicate primary and metastatic solid tumors, cancer stem cells, as well as microscopic tumor extensions [1]. The effectiveness of radiation to eliminate cancer cells is by directly interacting with critical cell targets [2] or indirectly by free radical production, the latter leading to increased apoptosis of cells [3]. One main challenge of traditional radiation therapy is that tumors can be located close to normal tissue or near organs at risk that can limit the amount of radiation dose delivered to the target volume [4]. Radiation sensitizers are agents that preferentially sensitize tissues and organs to ionizing radiation which have attracted tremendous attention recently [5]. On the other hand, radioprotectors have traditionally been used to reduce the complications associated with radiation therapy to normal tissues [6].

Nanomedicines have the potential to improve therapeutic benefits by specific tumor targeting due to their enhanced permeability and retention effect, though poor biocompatibility and target uptake can be potential issues [7]. Metal nanoparticles are good candidates for physical dose enhancements by inducing the production of augur electors and photoelectrons further leading to increased production of free radicals and increased by-stander effects [8]. Cerium oxide metal nanoparticles have unique properties that are advantageous in comparison to other nanoparticles. They have a crystal lattice consisting of a cerium core enveloped by an oxygen lattice and can coexist in Ce^3+^ and Ce^4+^ ions with the ability to have oxygen vacancies on their surface enabling modulation of free radicals according to the below redox chemistry [9].

$$CeO_{2}\;\;\;\overset{reduction}{\underset{oxidation}{⇄}}\;\;\;CeO_{2-\delta }+\frac{\delta }{2}O_{2}$$

Some studies have demonstrated a protective effect of nanoceria towards free radical induced damage [10,11] while others have shown increased induction of oxidative stress [12,13]. Environmental conditions like the pH appears to be a factor governing these paradoxical findings [12]. Studies of nanoceria in cancer cells have shown wide ranging effects, from anti-invasive properties [14] to simultaneous radio-sensitisation [15] and radioprotection [16]. Potential effects on health and environment reported include no negative effects [17,18] to adverse effects including kidney and lung damage [19,20]. *In vivo* effects of nanoceria may ultimately depend on their mode of use and more studies are needed to precisely characterize its localization, toxicity, and redox effects in human cells for taking this unique rare-earth element with promising characteristics for potential application in the clinics.

Our studies used a unique and relatively novel approach to localize unmodified and unlabeled cerium oxide nanoparticles in the triple negative breast cancer cell line, MDA MB231. Although, transmission electron microscopy is currently the gold standard for visualizing intracellular nanoparticles, the extensive sample preparation and difficult data interpretation owing to poor contrast arising from soft materials can be serious impediments for high-throughput studies [21]. An alternative approach is to use fluorescent tags, but this method can have some serious challenges including difficult labeling, potential alterations to the surface chemistry during labeling leading to altered bioactivity, label dissociation leading to erroneous results, low quantum efficiency and detection sensitivity, and photobleaching [22]. Since metallic nanoparticles provide excellent contrast with reflected light, we used reflective structured illumination microscopy without labeling the nanoparticles in conjunction with cell fluorescence imaging for effective compartmental visualization of nanoparticles.

We hypothesized that naked cerium oxide nanoparticles can be effectively localized in breast cancer tumor cells and the localized nanoceria will be safe for further biomedical applications. This hypothesis was tested in MDA MB231 cells using reflectance structured illumination imaging, flow cytometry, cell survival and reactive oxygen species assays.

## 2 Materials and methods

### 2.1 Raman spectroscopy

Raman spectroscopy was performed on a dried sample of Cerium dioxide nanoparticles (NP) using a Dilor XY micro-Raman spectrometer with a 532 nm laser and a 1200 groove/mm grating.

### 2.2 Culture of human breast cancer cells

The human adenocarcinoma cell line MDA MB231 (EGFR^+^ and ER^−^/PR^−^/HER2^−^ (i.e., triple negative)) was obtained from American Type Culture Collection (ATCC, Rockville, MD; product number: HTB-26). Cells were maintained in Leibovitz’s L-15 media (Corning, Manassas, VA) supplemented with 10% fetal bovine serum (FBS) (HyClone, GE Health Care Life Sciences, Marlborough, MA), and 1% penicillin-streptomycin (Gibco, Gaithersburg, MD) in a humidified incubator at 37 ^0^C. Cells were passaged every third day at approximately 80% confluency using a standard trypsin-EDTA (0.25%: 0.2%) protocol (Gibco, Gaithersburg, MD).

### 2.3 Reflectance structured illumination imaging of CeO_2_ nanoparticles *in vivo*

MDA MB231 cells were grown overnight on poly-D lysine coated 20 mm, no. 1 cover slips (Neuvitro, Vancouver, WA) in a 24-well plate (Corning, Manassas, VA) at a density of 2 x10^5^ cells/coverslip. Cells were treated with a colloidal dispersion of 10 to 20 nm cerium dioxide (30% colloidal suspension in water produced by Alfa Aesar, Ward Hill, MA) particles at a concentration of 50 μg/mL in complete L-15 media for 72 h. Cells were washed three times with 1X PBST, fixed with 4% paraformaldehyde for 10 min at room temperature, and then permeabilized with 0.2% triton X-100 for 10 min at room temperature. Cells were washed and then stained with Hoechst 33342 (Abcam, Cambridge, MA), mounted and sealed with nail polish. Slides were imaged with Reflectance Structured Illumination Microscopy (R-SIM) (Nikon Corp., Japan) with Orca Flash 4 Camera (Hamamatsu, Japan). To image with R-SIM, a half-mirror was placed in the light path instead of the dichroic and the configuration was set up in the fourth channel using 488 nm laser. The dichroic was set to BS20/80 with all light paths set to “through”. Images were processed and analyzed using Nikon Nis Elements 5.0 Imaging Software. Nanoparticles were displayed in red and cell nucleus in blue for reflectance images. Cells were shown in black and white for differential interference contrast images.

### 2.4 Dose dependent cellular uptake analysis of nanoparticles

The percentage of breast cancer cells taking up CeO_2_ NPs were assessed for various treatment concentrations of NPs. MDA MB231 cells were seeded overnight in a 24-well plate at a density of 2 x 10^5^ cells/well. Cells were treated with either 0, 25, 50, 100, or 200 μg/mL of CeO_2_ NPs in 2 mL L-15 complete media for 72 h. Cells were washed three times with 1X PBST and the attached cells were collected by centrifugation and analyzed using FACSClibur flow-cytometer (Becton Dickinson, Franklin Lakes, NJ). The dose dependent cellular uptake of nanoparticles was visualized using side scatter (SSC–H) and the percentage of cells taking up the NP was calculated with FlowJo (Becton Dickinson, Ashland, OR).

### 2.5 Cell survival analysis

The percentage viable, early and late apoptotic, and necrotic cells were assessed using Annexin-V-FITC (AV) and Propidium Iodide (PI). MDA MB231 cells were seeded overnight in a 24-well plate at a density of 0.1 x 10^6^ cells/well. Cells were treated with either 0, 25, 50, 100, or 200 μg/mL of CeO_2_ NPs in 2 mL L-15 complete media for 72 h. The attached and floating cells were collected by centrifugation and resuspended in 100 μL of binding buffer containing 5 μL AV and 5 μL PI according to manufacturer’s instructions. After the incubation period (15 min at room temperature), cells were centrifuged and the pellet was resuspended in 200 μL of binding buffer. Cells were stored on ice and flow cytometry analysis of the AV/PI was performed within 1 h. The fluorescence intensities (green BL1-H and red YL2-H) were measured using flow cytometry. In each sample, an average of 5,000 cells were recorded (gated to exclude cell debris), and the percentages of viable (AV^-^/PI^-^), early apoptotic (AV^+^/PI^-^), apoptotic and necrotic (AV^+^/PI^+^), and already dead (AV^-^/PI^+^) cells were analyzed with FlowJo.

### 2.6 Reactive oxygen species analysis

Intracellular reactive oxygen species (ROS) levels were measured using the peroxide-dependent oxidation of dihydrorhodamine 123 (DHR123), to the fluorescent compound, rhodamine 123. MDA MB231 cells were seeded overnight in a 24-well plate at a density of 0.2 x 10^6^ cells/well. Cells were treated with either 0, 25, 50, 100, or 200 μg/mL of CeO_2_ NPs in 2 mL L-15 complete media for 72 h. Adherent cells were harvested by trypsinization and incubated in 500 μL of medium containing 10 μM DHR for 30 min at 37 °C in the dark. Cells were then washed twice in 1X PBS and DHR green fluorescence was analyzed by flow-cytometry at FL1-H channel using an excitation wavelength of 488 nm and an emission of 530/30. The Median fluorescence intensity (MFI) was assessed after correcting for autofluorescence.

### 2.7 Statistical analysis

At least three experiments were done for each experiment. Bar graphs indicate averages of the three experiments, and error bars represent standard deviation between experiments. Statistical significances between treatments under test conditions were compared using Prism 8 software (Graphpad, San Diego, CA). P-values were determined using one-way analysis of variance (ANOVA), Tukey’s post hoc test, and considered significant for p < 0.05.

## 3 Results and discussion

### 3.1 The localization of pure unlabeled CeO_2_ nanoparticles in MDA MB231 cells is confirmed using Raman spectroscopy and Reflectance structured illumination imaging

**Fig. 1:**
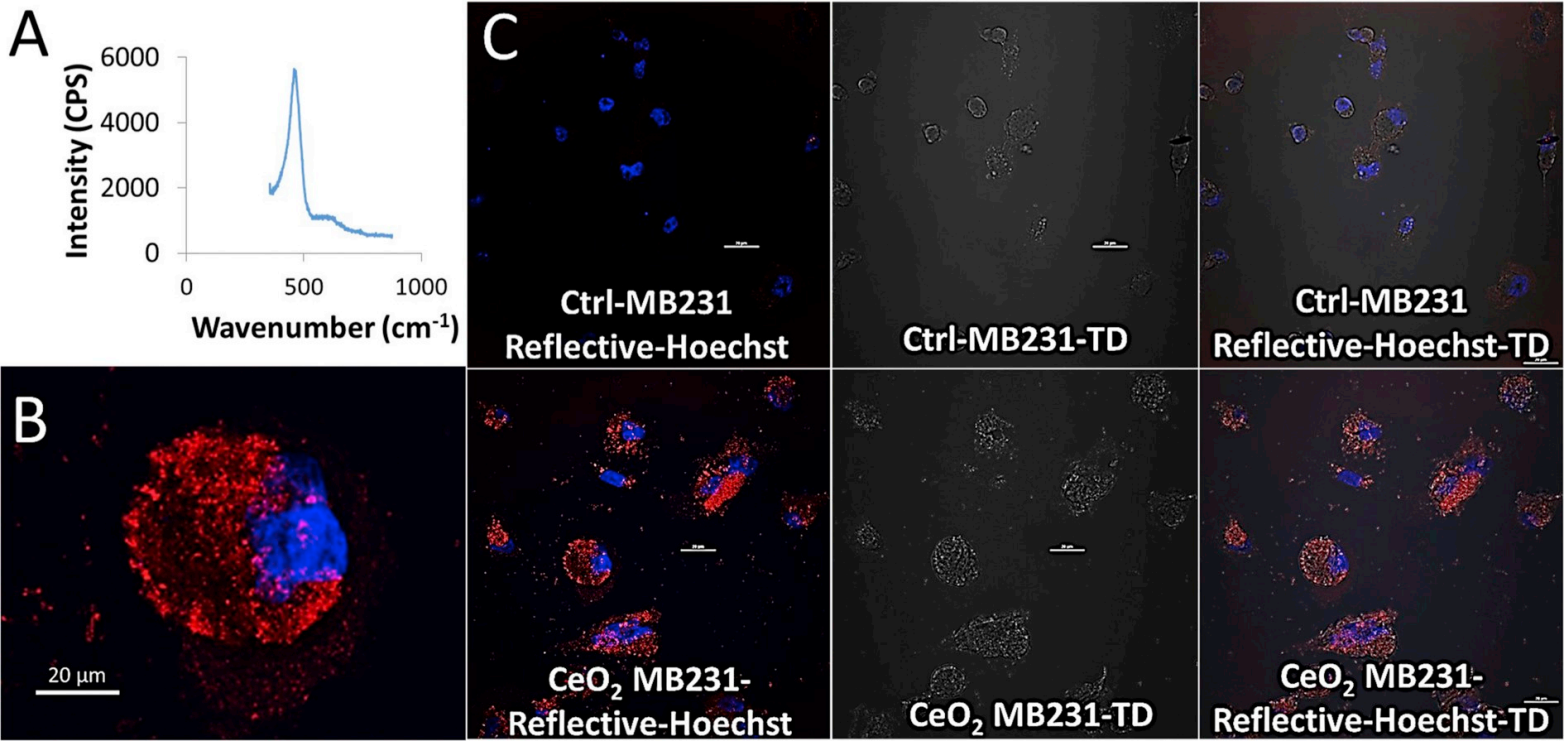
Characterization and visualization of CeO_2_ nanopartcles is MDA MB231 breast cancer cells. (A) Raman spectroscopy at room temperature of CeO_2-__δ_ sample. The prominent band in the Raman spectrum is active triply degenerate. Software zoomed (B) and comparison reflectance structured illumination images along with corresponding differential interference contrast images of nanoceria in MBA MB231 cells labelled with Hoechst 33342 (C).

We first evaluated the chemical identity of the nanoparticles and whether its uptake can be detected easily in cells without any labeling modifications. Raman spectroscopy was performed to identify the chemical signature of the nanoparticles (Fig. 1A) and reflectance structured illumination imaging was done to confirm the presence of nanoparticles within the cells (Fig. 1B and C). Raman spectroscopy revealed a main band around 460 cm^-1^ corresponding to pure ceria, which is the triply degenerate symmetric breathing F_2g_ mode characteristic of the stretching vibration of Ce–O in the O_h_ point group within the fluorite-type cubic crystal structure of CeO_2_. Reflectance imaging revealed the presence of copious amounts of ceria nanoparticles within the cytoplasm and along the cell membrane (Fig. 1B, Fig. 1C Lower panel), while only the cell nucleus was visible in the untreated control cells (Fig. 1C Upper Panel). This demonstrated that reflectance imaging technique can be effectively used to visualize the presence of metal oxide nanoparticles within cells without any attached fluorophores. A comparison of differential interference contrast images of both treated and untreated samples did not show any significant visual differences (Fig. 1C Middle panel) indicating that transmitted light is not capable of delineating the nanoparticles. An overlay of the reflected and transmitted images showed that the nanoparticles were localized within the cells (Fig. 1C Right lower panel) while no corresponding visualization was obtained with the control sample overlays (Fig. 1C Right upper panel). Overall, we were able to successfully confirm the localization of ceria nanoparticles within breast cancer cells using these techniques.

### 3.2 CeO_2_ nanoparticles were taken up in a dose-depended manner by more cells and in increasing quantities

**Fig. 2:**
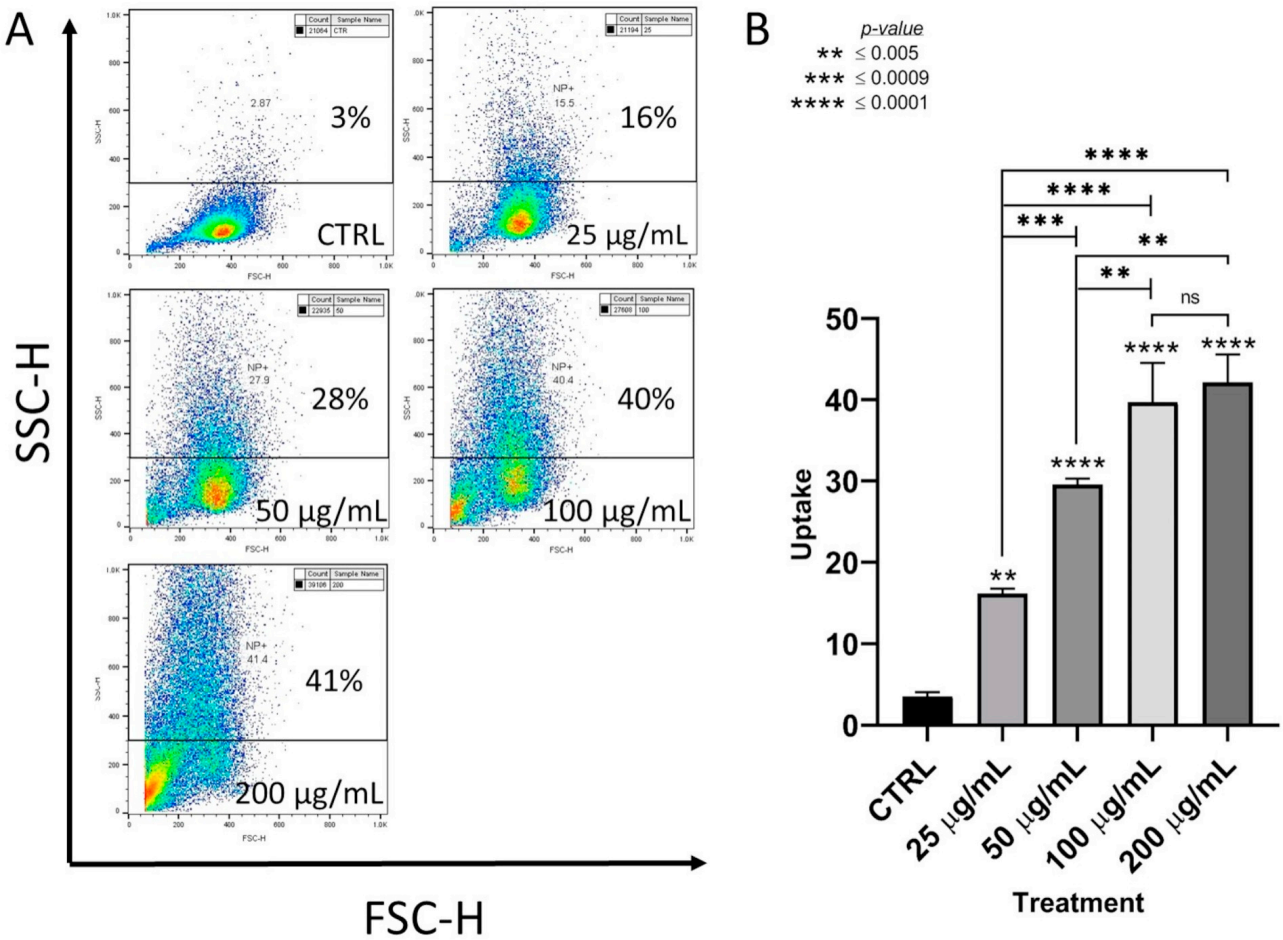
Dose-dependent uptake of CeO_2_ nanoparticles in MBA MB231 cells. Analysis was performed on untreated and breast cancer cells treated with 25, 50, 100, and 200 μg ml^-1^ nanoceria. The side scatter population of the bivariate plot was gated to isolate the cells taking up the nanoparticles (A) and a one-way ANOVA was used to compare the percentage uptake for each treatment concentration (B).

In order to quantify the ability of a population of MDA MB231 cells to take up CeO_2_ nanoparticles, cells were not treated or treated with increasing concentrations of nanoceria and analyzed by flow cytometry. The resultant bivariate plot gated for side scatter (SSC–H) showed 3%, 16%, 28%, 40%, and 41% of cells taking up the nanoparticles, when treated with 0, 25, 50, 100, and 200 μg/mL of nanoceria, respectively (Fig. 2A). An increase in signal for the gated population was also noted in proportion to the increasing concentration of nanoceria treatment. The amount of nanoparticle uptake was significant at all doses compared to the untreated sample, as well as when compared among the samples, except between 100 and 200 μg/mL concentrations (Fig. 2B). These results indicated that at higher nanoceria concentrations more number of breast cancer cells were taking up the nanoparticles and also more nanoparticles got localized in cells in proportion to treatment concentrations. This is significant when considering the use of nanoceria as a radiosensitizer for radiation therapy applications.

### 3.3 The intracellular presence of CeO_2_ nanoparticles had no adverse effects on the health of the cells

**Fig. 3:**
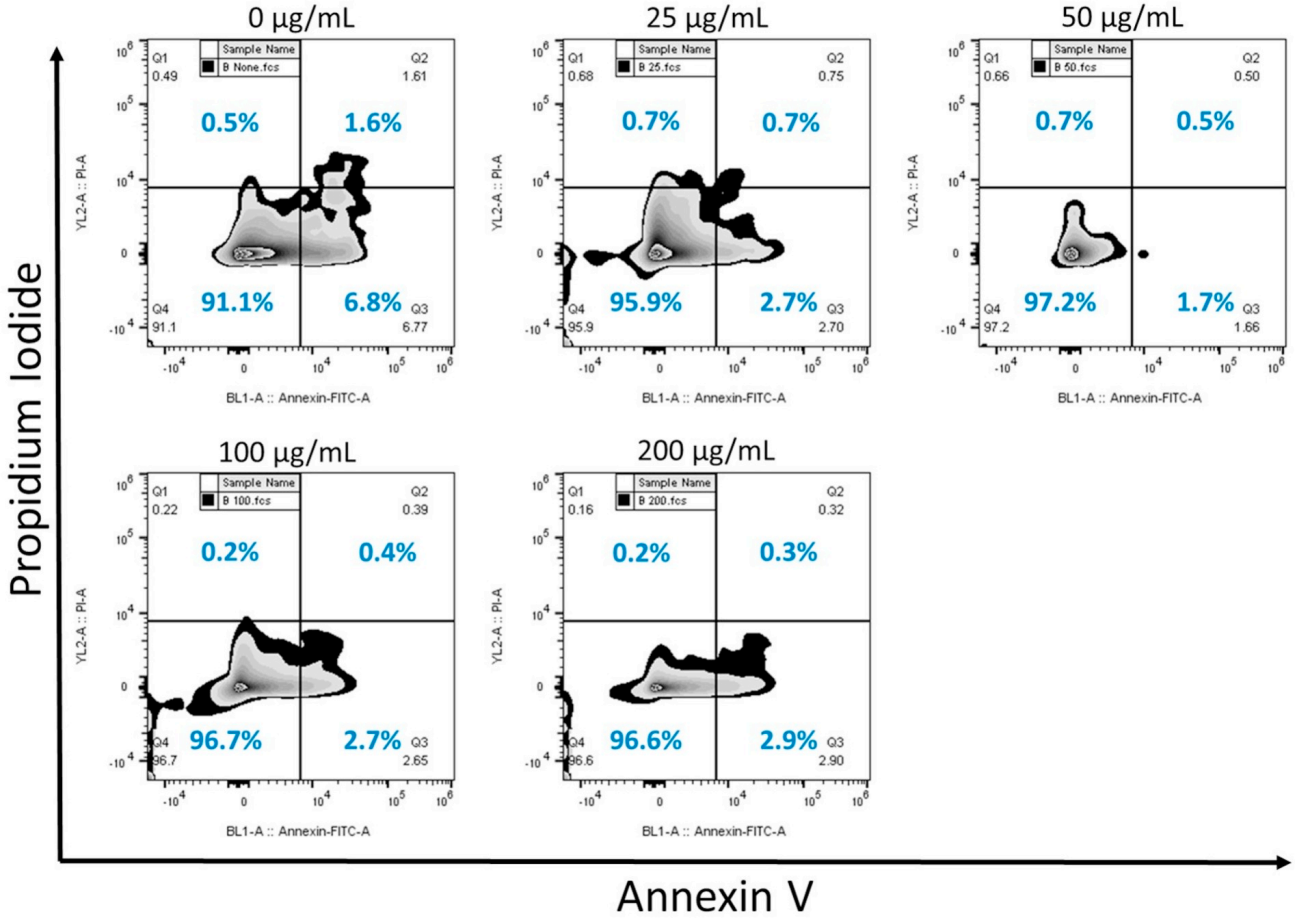
Intracellular CeO_2_ caused no adverse effects on MBA MB231 cell survival. Untreated and breast cancer cells treated with 25, 50, 100, and 200 μg ml^-1^ nanoceria were analyzed with quadrant gating for green fluorescence (Annexin V, FL1-A) and red fluorescence (Propidium Iodide, FL2-A). Zebra plots were used for a mix of contour (20%) and density plots to represent the area distribution of cells.

To investigate the potential of nanoceria to induce cell death, MDA MB231 cells, treated with increasing concentrations of CeO_2_ nanoparticles, were subjected to Annexin V/ Propidium Iodide staining and analysis by flow cytometry. The summary of results are shown in Fig. 3. There were no significant changes in overall cell survival at any treatment concentrations. A slight increase in the healthy population was noted with increasing nanoparticle concentrations, along with a decrease in early apoptotic cells at all treatment concentrations. These results showed that pure cerium oxide nanoparticle had no toxic effects on MDA MB231 cells at the given concentrations. Our experiments indicated the potential for the use of cerium oxide nanoparticles safely *in vivo*.

### 3.4 The intracellular presence of CeO_2_ nanoparticles significantly reduced ROS levels in MDA MB231 cells in a dose-dependent manner

**Fig. 4:**
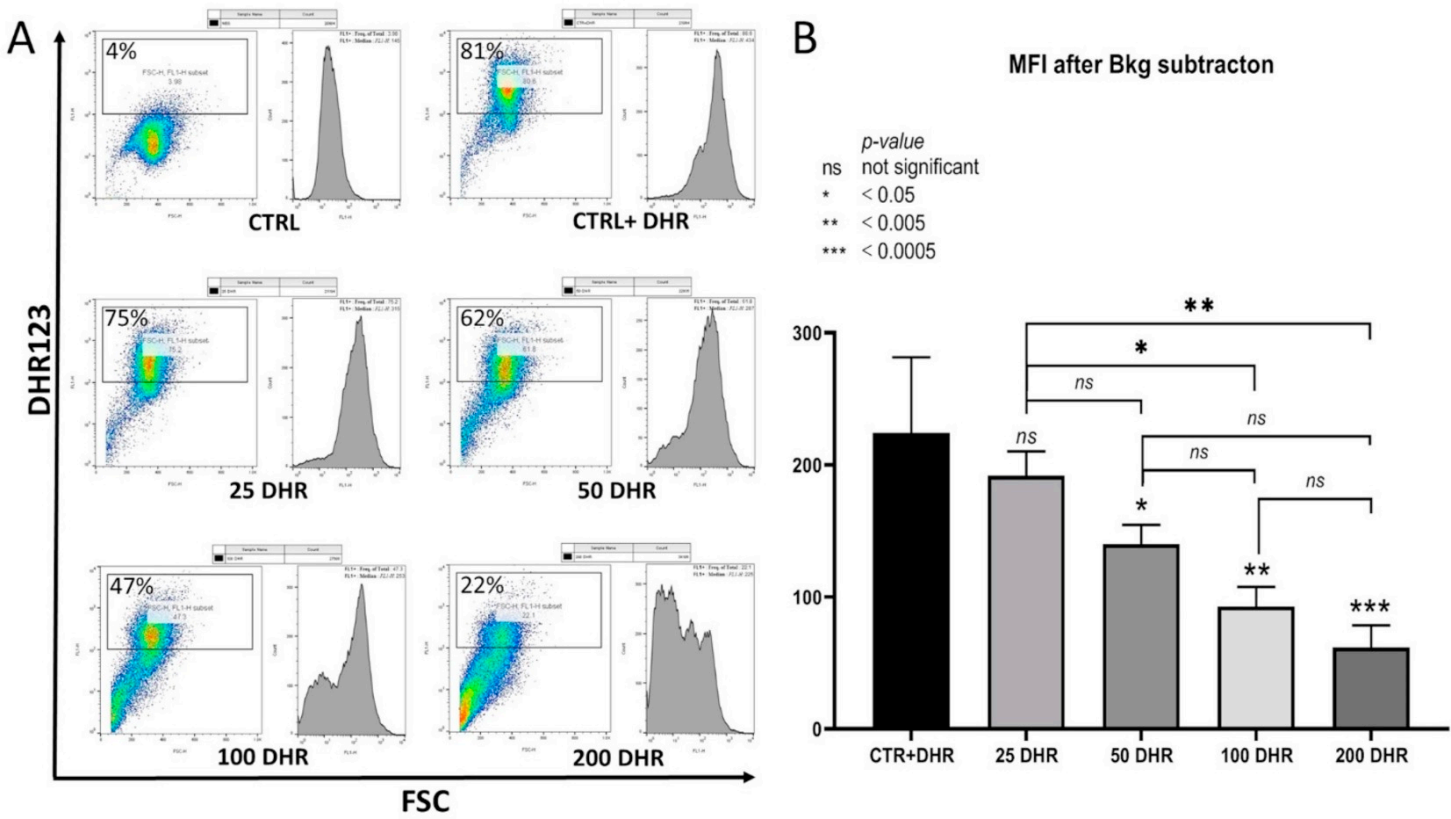
Intracellular CeO_2_ nanoparticles downregulate ROS levels in MDA MB231 cells. Flow-cytometric profiles of untreated-unlabeled cells, and untreated, 25, 50, 100, 200 μg ml^-1^ nanoceria-treated cells labelled with 10 μM DHR123 (A). A one-way ANOVA was used to compare background-subtracted median fluorescent intensities in the FL1 subpopulation of different treatment samples.

In order to examine the possible effects of intracellular cerium oxide nanoparticles on redox systems in MDA MB231 cells, we measured the levels of rhodamine 123 as an indicator of oxidation of dihydrorhodamine 123 by peroxynitrite anion [ONOO]^-^ and hydrogen peroxyde anion [HOO]^-^ species in CeO_2_ nanoparticle-treated cells relative to untreated cells. The FL1 population was gated to include green fluorescent cells with a plot of the median fluorescent intensity (Fig. 4A). The percentage fluorescent population detected were 81%, 75%, 62%, 47%, and 22% for samples treated with 0, 25, 50, 100, and 200 μg/mL of nanoceria, respectively. The background autofluorescence was 4%. An analysis of the background corrected median fluorescence intensities showed that there was significant dose-dependent reduction of MFI among all the nanoceria-treated samples in comparison to the untreated sample (Fig. 4B). There was significant reduction in ROS for 100 and 200 μg/mL treatments compared to the 25 μg/mL treatment, however, no significant differences were noted among higher treatment samples (50, 100, and 200 μg/mL). These results indicated that cerium oxide nanoparticles significantly downregulated the production of reactive oxygen species in a dose dependent manner in MDA MB231 cells.

In conclusion, we have shown for the first time that pure cerium oxide nanoparticles can be localized relatively easily in breast cancer cells using reflectance confocal imaging. These cells can take up higher amounts of nanoceria without any adverse effects to cellular health by significantly downregulating reactive oxygen species levels, thus paving the way for its safe use as a potential radiosensitizer *in vivo* for the treatment of breast cancer.
